# ROS signaling by NADPH oxidase 5 modulates the proliferation and survival of prostate carcinoma cells

**DOI:** 10.1002/mc.22255

**Published:** 2015-01-05

**Authors:** Monika Höll, Rafal Koziel, Georg Schäfer, Haymo Pircher, Alexander Pauck, Martin Hermann, Helmut Klocker, Pidder Jansen‐Dürr, Natalie Sampson

**Affiliations:** ^1^ Institute for Biomedical Aging ResearchUniversity of InnsbruckInnsbruckAustria; ^2^ Tyrolean Cancer Research InstituteMedical University of InnsbruckInnsbruckAustria; ^3^ Department of UrologyMedical University of InnsbruckInnsbruckAustria; ^4^ Department of Anaesthesiology and Critical Care MedicineMedical University of InnsbruckInnsbruckAustria

**Keywords:** NOX, prostate cancer, reactive oxygen species, protein kinase C, c‐Jun N‐terminal kinase

## Abstract

Prostate cancer (PCa) is the most commonly diagnosed cancer and second leading cause of male cancer death in Western nations. Thus, new treatment modalities are urgently needed. Elevated production of reactive oxygen species (ROS) by NADPH oxidase (Nox) enzymes is implicated in tumorigenesis of the prostate and other tissues. However, the identity of the Nox enzyme(s) involved in prostate carcinogenesis remains largely unknown. Analysis of radical prostatectomy tissue samples and benign and malignant prostate epithelial cell lines identified Nox5 as an abundantly expressed Nox isoform. Consistently, immunohistochemical staining of a human PCa tissue microarray revealed distinct Nox5 expression in epithelial cells of benign and malignant prostatic glands. shRNA‐mediated knockdown of Nox5 impaired proliferation of Nox5‐expressing (PC‐3, LNCaP) but not Nox5‐negative (DU145) PCa cell lines. Similar effects were observed upon ROS ablation via the antioxidant N‐acetylcysteine confirming ROS as the mediators. In addition, Nox5 silencing increased apoptosis of PC‐3 cells. Concomitantly, protein kinase C zeta (PKCζ) protein levels and c‐Jun N‐terminal kinase (JNK) phosphorylation were reduced. Moreover, the effect of Nox5 knockdown on PC‐3 cell proliferation could be mimicked by pharmacological inhibition of JNK. Collectively, these data indicate that Nox5 is expressed at functionally relevant levels in the human prostate and clinical PCa. Moreover, findings herein suggest that Nox5‐derived ROS and subsequent depletion of PKCζ and JNK inactivation play a critical role in modulating intracellular signaling cascades involved in the proliferation and survival of PCa cells. © 2014 The Authors. *Molecular Carcinogenesis* published by Wiley Periodicals, Inc.

**Abbreviations**
cPDL: cumulative population doubling
H_2_O_2_: hydrogen peroxide
IHC: immunohistochemistry
JNK: c‐Jun N‐terminal kinase
NAC: N‐acetylcysteine
Nox: nicotinamde adenine dinucleotide phosphate oxidase
PCa: prostate cancer
PKC: protein kinase C
PSA: prostate‐specific antigen
qPCR: quantitative real‐time PCR
RabMab: rabbit monoclonal antibody
ROS: reactive oxygen species
shRNA: small‐hairpin RNA
TMA: tissue microarray

## INTRODUCTION

Prostate cancer (PCa) is the most commonly diagnosed cancer in men and second leading cause of male cancer death in Western societies 1. Initial systemic treatment of advanced PCa targets androgen signaling/metabolism 2. Despite initial tumor regression in approximately 90% of patients, many patients succumb to castration‐resistant PCa within 3 years 3. Thus, a better understanding of the molecular mechanisms underlying PCa development and progression are required to develop more effective treatment strategies. NADPH oxidases (Nox) are transmembrane proteins that transport electrons across biological membranes thereby generating reactive oxygen species (ROS). The Nox family comprises seven members, namely Nox1–5, Duox1, and Duox2 4. ROS are highly reactive metabolites that interact with a number of molecules irreversibly altering their function and thereby inducing cellular damage 4. For example, increased ROS production in human endothelial cells leads to premature senescence 5 whereas reducing ROS production by knocking down expression of Nox4 extends the replicative lifespan of these cells 6. However, ROS also play an important role as second messengers in fundamental cellular processes such as proliferation, differentiation and apoptosis in particular by modulating signal transduction pathways via reversible oxidation of redox‐sensitive proteins 7. Thus, ROS produced by Nox enzymes can have both pro‐ and anti‐proliferative capacities 7 with enhanced ROS production reported to promote cell proliferation and survival in various cancer types, including glioma, melanoma, renal cell carcinoma, pancreatic cancer, and thyroid cancer 8, 9, 10, 11, 12.

Several studies have also implicated a role of Nox‐derived ROS in prostate cancer. For example, compared to normal prostatic cells, PCa cells exhibit increased extra‐mitochondrial ROS production, which correlates with a highly invasive and metastatic phenotype 13, 14. Accordingly, human prostate tumors show increased ROS levels 14, 15. Due to the limited availability of specific antibodies to individual members of the Nox family, it currently remains unclear which Nox isoforms are expressed in the prostate in vivo and which isoforms contribute to the pathogenesis of PCa. Nonetheless, specific antisense targeting of Nox enzymes was shown to inhibit cell proliferation, migration and metastatic potential of PCa cells 16, 17. Similarly, knockdown of p22(phox), an accessory subunit critical for the function of Nox enzymes, suppressed angiogenesis and growth of prostate xenografts in mice 18. In addition, Nox4‐derived ROS were shown to be essential for the development of a reactive stromal phenotype in vitro, which promotes the development and progression of PCa 19. Recently, Nox4 was identified as part of a 36‐gene signature that predicts clinical outcome within the subset of prostate tumors harboring a TMPRSS2‐ERG gene rearrangement 20. Nox4 was shown to be elevated in those patients of this subset who experience biochemical recurrence 20, 21. Collectively, these findings suggest that elevated ROS production by Nox family members contributes to the development and progression of PCa. As such, targeting Nox enzymes may represent an effective anti‐cancer therapy for PCa. Indeed Nox1 activation by parthenolide sensitized PC‐3 cells to radiation treatment 22 and androgen deprivation therapy of 22Rv1 PCa cells, in which Nox2 and Nox4 mRNAs are androgen‐dependently regulated, sensitized these cells to radiation treatment 23. Together these data suggest that modulating the Nox‐dependent intracellular redox status alone or in combination with current treatment strategies may offer enhanced therapeutic benefit for PCa patients.

To address the role of Nox family members in PCa, we queried the Human Protein Atlas, analyzed Nox/Duox mRNA levels via gene expression microarrays on benign and malignant prostate tissue specimens and screened a panel of commonly used PCa cell lines for the expression of Nox/Duox genes. These studies identified Nox5 as an abundantly expressed Nox isoform in the PCa cell lines examined with high levels of Nox5 protein in the prostatic epithelium in a set of clinical specimens derived from well‐characterized PCa patients. Subsequent functional analyses indicate that Nox5 plays a critical role in regulating the proliferation and survival of PCa cells.

## MATERIALS AND METHODS

### Cell Culture

Malignant (LNCaP, VCaP, PC‐3, and DU145) and benign (RWPE1) prostate cell lines were purchased from American Type Culture Collection (ATCC; Rockville, MD). The human telomerase reverse transcriptase‐immortalized benign prostate epithelial cell line EP156T has been described previously 24. Cells were maintained according to the distributor's instructions at 37°C in a humidified atmosphere with 5% CO_2_, as described 25. For production of lentiviral particles, HEK293‐T cells were maintained in DMEM containing 2 mM l‐glutamine, 100 units/mL penicillin, 0.1 mg/mL streptomycin and 10% (w/v) fetal bovine serum (heat inactivated).

### Lentiviral Infection

Production of lentiviral particles was carried out according to the manufacturer's protocol (Addgene; Cambridge, MA) using the packaging plasmids pMD2.G and psPAX2 (both from Addgene) and the lentiviral vector pLKO.1 containing Nox5‐specific (TTCTATCGGAGTCAAATAGGG) or scrambled control (CCGCAGGTATGCACGCGT) small hairpin RNA (shRNA) (Thermo Fisher Scientific; Waltham, MA). For lentiviral infection, 100,000 cells were seeded in 6‐well plates. The next day, culture medium containing lentiviral particles to the amount of 4 MOI (multiplicity of infection) and 8 μg/mL hexadimethrine bromide (Sigma–Aldrich; St. Louis, MO) were added to the cells. 24 h after infection the medium was changed. Puromycin selection (500 ng/mL) was started 48 h after infection, as described 6.

### Illumina Gene Expression Arrays

mRNA expression levels of Nox/Duox isoforms in benign and malignant tissue of radical prostatectomy specimens were extracted from a global gene array expression analysis performed with macro‐dissected tissue samples using Illumina Human Sentrix‐12 BeadChip arrays (Illumina, San Diego, CA) as described 26. The data sets have been deposited in the NCBI Gene Expression Omnibus database (GSE32571, GSE32873, and GSE36531).

### PCR‐Based Quantification of mRNA Levels

For quantitative real‐time PCR (qPCR), total RNA was isolated from cells using the RNeasy Mini kit (Qiagen; Hilden, Germany). cDNA was synthesized from total RNA templates (1 μg) using the RevertAid First Strand cDNA Synthesis kit (Thermo Fisher Scientific) and oligo (dT) primers 6. Primers for detection of mRNA were as follows: 5′‐TTCACCAATTCCCAGGATTGAAGTGGATGGTC‐3′ (forward) and 5′‐GACCTGTCACGATGTCAGTGGCCTTGTCAA‐3′ (reverse) for Nox1; 5′‐GTCACACCCTTCGCATCCATTCTCAAGTCAGT‐3′ (forward) and 5′‐CTGAGACTCATCCCAGCCAGTGAGGTAG‐3′ (reverse) for Nox2; 5′‐ATGAACACCTCTGGGGTCAGCTGA‐3′ (forward) and 5′‐GGATCGGAGTCACTCCCTTCGCTG‐3′ (reverse) for Nox3; 5′‐AGTCCTTCCGTTGGTTTG‐3′ (forward), and 5′‐AAAGTTTCCACCGAGTAC‐3′ (reverse) for Nox4; 5′‐CCCTTTGCTTCCATTCTG‐3′ (forward) and 5′‐TCACAAACCACTCGAAAGAC‐3′ (reverse) for Nox5; 5′‐ATGTGCCAGATACCCAAAGC‐3′ (forward) and 5′‐CAGCTGACGGATGACTTGAA‐3′ (reverse) for Duox1; 5′‐AAAGGCTCCCCAGAGGATAA‐3′ (forward) and 5′‐CTCCCGGAACATAGACTCCA‐3′ (reverse) for Duox2; 5′‐CAGTCTGCGGCGGTGTT‐3′ (forward) and 5′‐GCAAGATCACGCTTTTGTTCCT‐3′ (reverse) for PSA as well as 5′‐GAATTCACCCCCACTGAAAA‐3′ (forward) and 5′‐CTCCATGATGCTGCTTACA‐3′ (reverse) for the “housekeeping” gene B2M, which served as normalization standard. The cDNA equivalent of 50 ng RNA was applied to PCR amplification in combination with the LightCycler™ 480 SYBR Green I Master Mix (Roche Diagnostics GmbH, Vienna, Austria). qPCR was performed in triplicates on a LightCycler 480 II Real‐Time PCR Detection System (Roche Diagnostics GmbH) in a final reaction volume of 20 μL per well, as described 19. Fold changes in gene expression were calculated as described 27.

### Protein Detection by Immunoblot Analysis

Western blotting of total cell lysates and membrane lysates for Nox detection was performed as described 6. Briefly, cells were trypsinized, washed once in cold PBS, shock‐frozen in liquid nitrogen and thawed on ice. Samples were then sonicated, centrifuged and the supernatant was mixed with standard SDS/PAGE sample buffer and denoted as soluble fraction. The insoluble pellet was resuspended in membrane protein extraction buffer, sonicated, and centrifuged as described before. Protein concentration was assessed as for total cell lysates, the supernatant (membrane fraction) was mixed with an equal volume of membrane protein sample buffer, incubated at 40°C for 30 min, subjected to SDS/PAGE (10–15% gels) and transferred to PVDF membranes. Membranes were blocked with 5% BSA in TBS (50 mM Tris–HCl and 150 mM NaCl)/0,1% Tween 20 and incubated with primary antibodies overnight. After incubation with horseradish peroxidase‐conjugated secondary antibodies (Dako Cytomation; Copenhagen, Denmark 1:5000), proteins of interest were visualized with ECL™ Western Blotting Detection Reagent (GE Healthcare; Little Chalfont, United Kingdom). Primary antibodies used were as follows: Nox5 (gift from KH Krause, Geneva, 1:500), p38 MAPK, phospho‐p38 MAPK, Akt, phospho‐Akt, phospho‐p44/42 MAPK (Cell signaling; Danvers, MA, 1:1000), JNK1/3, p‐JNK, PKCζ (Santa Cruz, 1:1000), PKCε, ERK1 (Santa Cruz, 1:500), β‐actin, α‐tubulin (Sigma; St. Louis, MO, 1:1000–1:10,000), p84 (Abcam; Cambridge, United Kingdom, 1:1000). RabMab 27–6, a rabbit monoclonal antibody to Nox4, was generated by immunizing rabbits with the C‐terminal fragment of Nox4 (355–578) after recombinant expression in *E.coli*. In collaboration with a commercial provider (Epitomics Inc., Buringame, CA), rabbit hybridoma were derived and tested for Nox4 detection in Western blot, yielding rabbit monoclonal antibody (RabMab) 27–6. RabMab 27–6 was used at 1:500 dilution.

### Determination of Cumulative Population Doublings (cPDL)

A total of 200 000 cells were seeded in 6‐well plates and passaged every three to four days. PC‐3 cells infected with the lentiviral vector pLKO.1 containing Nox5‐specific shRNA or control shRNA were grown under puromycin selection. PC‐3 wild type cells were treated with N‐acetylcysteine (6 mM and 10 mM) or with the JNK inhibitor SP600125 (1 μM and 7 μM; Tocris Bioscience, Ellisville, MO). cPDL were estimated using the following equation: *n* = (log_10_ *F*−log_10_ *I*)/0.031 where *n* is the population doubling, *F* the number of cells at the end of one passage and *I* the number of cells that were seeded at the beginning of one passage 6. cPDL were counted over a period of 30–35 days. Single days were chosen for bar graphs, which represent mean values of three independent experiments.

### Bromodeoxyuridine (BrdU) Staining for Quantification of Cell Proliferation

DNA synthesis was assessed using the 5‐bromo‐2′‐deoxyuridine Labeling and Detection Kit I (Roche Applied Science, Vienna, Austria) according to the manufacturer's instructions for adherent cells. After the staining procedure, coverslips were analyzed by fluorescence microscopy, as described 6. Cells of three visual fields were counted and the number of BrdU‐positive cells was expressed as percentage of total cell number.

### Caspase‐Glo^®^3/7 Assay

To address caspase 3‐ and 7 activity, a Caspase‐Glo^®^3/7 Assay (Promega; Madison, WI) was performed following the manufacturer's instructions. Briefly, 18 000 PC‐3 scrambled or Nox5 knockdown cells were seeded in a 96 well plate in 100 μL DMEM the day before the experiment to reach a confluency of approximately 90%. As positive control, PC‐3 scrambled were pre‐treated with staurosporine 1 μM for 4.5 h at 37°C. After incubation, 100 μL of caspase 3/7 reagent containing buffer and substrate were added to each well, mixed and incubated for 1 h at room temperature in the dark. Luminescence was measured with the multi‐label reader Victor X5 (Perkin Elmer; Waltham, MA) and caspase activity was expressed in relative light units (RLU) 28. Luminescence was normalized to cell titer using a CellTiter‐Glo® Luminescent Cell Viability Assay (Promega).

### Determination of Mitochondrial and Cytosolic H_2_O_2_ Levels

For the detection of mitochondrial or cytosolic H_2_O_2_, we used the HyPer reporter protein system from Evrogen (Moscow, Russia). This system comprises two different expression vectors coding either for an untagged HyPer protein (HyPer‐dCyto) or a tagged HyPer protein containing two tandemly arranged mitochondrial targeting sequences in frame with the HyPer cDNA (HyPer‐dMito) (http://www.evrogen.com/products/HyPer/HyPer.shtml), which is recognized by the mitochondrial import complex and imported into the mitochondria. Cells were transfected with control, pHyPer‐dMito or pHyPer‐dCyto plasmids 29, using Lipofectamine® 2000 Reagent (Invitrogen, Carlsbad, CA). After 24 h live cells were analyzed by confocal microscopy. As a positive control, cells were pre‐incubated for 30 min with 250 μM H_2_O_2_. Cell nuclei were counterstained 30 min before imaging with 10 μg/mL Höchst 33258 (Invitrogen).

### Generation of Cell Clots for Immunohistochemistry

A total of 5 × 10^6^ cells were resuspended in 100 μL PBS supplemented with Mg^2+^ and Ca^2+^. 150 μL EDTA‐plasma and 150 μL thrombin were added to the cell suspension. The suspension was mixed carefully and incubated for 10 min at room temperature to allow coagulation. Cells clots were placed in 4% formaldehyde over‐night for fixation and afterwards embedded in paraffin. Slices were cut using a microtome and fixed on an object plate for immunohistochemical staining.

### Immunohistochemistry (IHC)

For IHC evaluation of Nox5 protein abundance in benign prostate and prostate tumor tissue, a tissue microarray (TMA) comprising 192 tissue cores of 48 cases (3 tumor and 1 benign cores per case) was immunostained, of which 44 cases could be evaluated. Paraffin‐embedded primary tumor specimens were obtained from previously untreated patients who had undergone radical prostatectomy at the Department of Urology, Innsbruck Medical University after prostate cancer diagnosis in a PSA early cancer detection program 30. Use of patients’ samples was approved by the ethics committee of the Innsbruck Medical University. IHC analysis was performed with 5 μm TMA sections on a Ventana Discovery‐XT staining automated system (Roche, Mannheim, Germany). Standard CC1 (Tris‐borate pH 7.8, 48 min at 98°C) pre‐treatment for antigen retrieval was followed by incubation for 1 h with a rabbit anti‐Nox5 polyclonal antibody (Atlas HPA019362) diluted 1:250 in Ventana Antibody Diluent followed by secondary universal antibody solution for 30 min, staining with DAP map kit and counter staining for 4 min with hematoxylin (HE) II bluing reagent (all IHC reagents from Roche). Specificity of staining was controlled by including a control antibody (Dako). Consecutive sections were HE stained or stained for the tumor marker AMACR and the non‐malignant benign gland specific marker P63 for correct assignment of non‐malignant and cancer areas. Nox5 immunostaining was evaluated by an experienced uropathologist (G.S.) using a quick score system that combines the number of positive cells and mean staining intensity to generate a score between 0 and 12.

### Confocal Laser Scanning Microscopy

Confocal microscopy was performed with a spinning disk confocal system (UltraVIEW VoX; Perkin Elmer, Waltham, MA) connected to a Zeiss AxioObserver Z1 microscope (Zeiss, Oberkochen, Germany). Images were acquired with Volocity software (Perkin Elmer; Waltham, MA) using a 63× oil immersion objective with a numerical aperture of 1.42. Images shown are z‐stacks of 5 planes with a spacing of 1 μm. Surface blots were illustrated using Image J software.

### Statistics

All numerical data are presented as mean ± SEM from at least three independent experiments or several parallel measurements (indicated in figure legends). When reasonable, values are shown relative to controls, which were set to 100%. Student's *t*‐test was used to compare groups and differences where *P* < 0.05 were considered statistically significant. Statistically significant differences are denoted ns, not significant where *P* > 0.05; *P < 0.05; **P < 0.01; ***P < 0.001.

## RESULTS

### Nox5 is Expressed in Glandular Epithelial Cells of the Benign and Malignant Prostate

**Figure 1:**
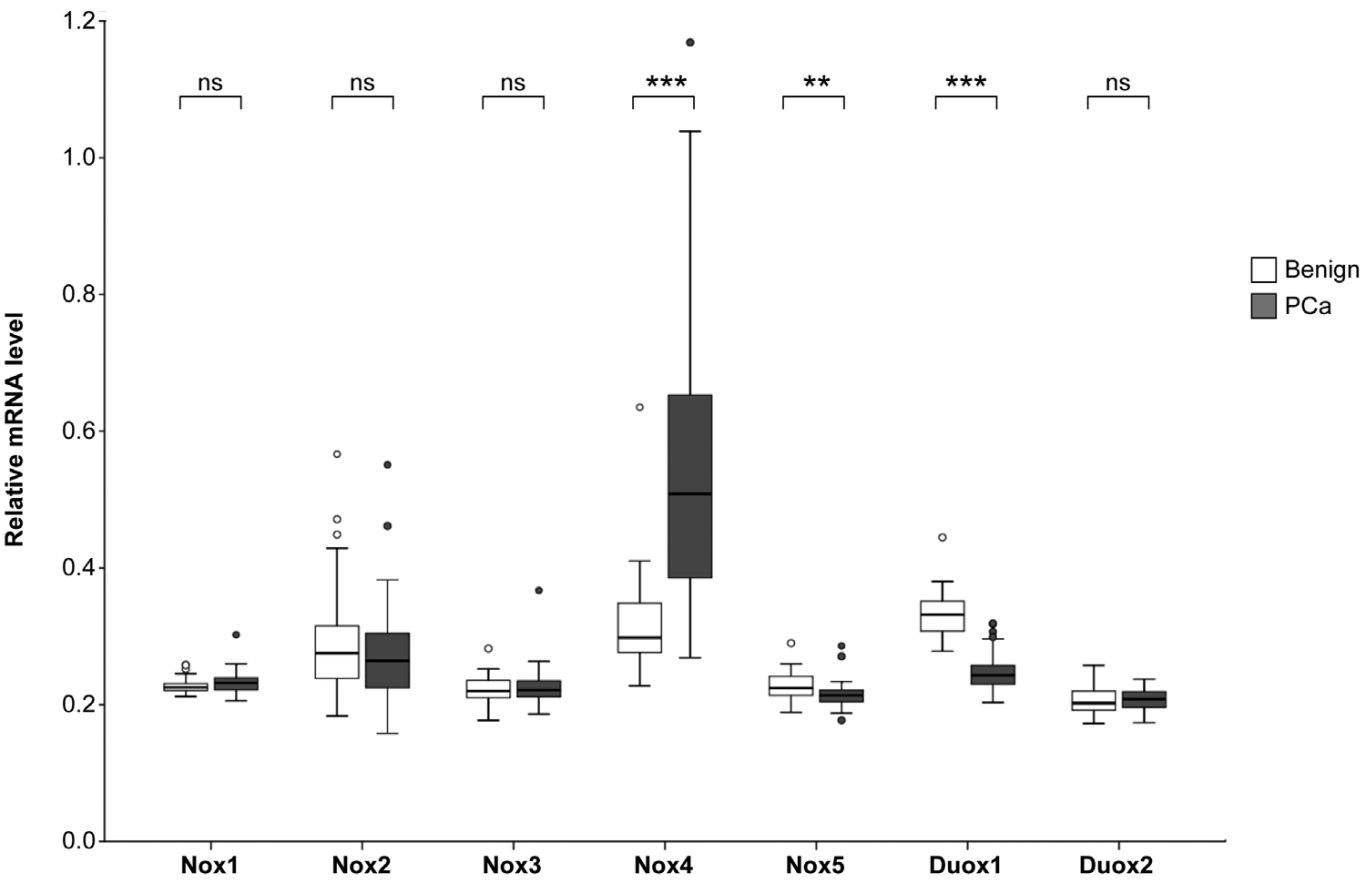
Expression of Nox and Duox isoforms in the benign and malignant prostate. Gene expression levels of Nox/Duox isoforms were determined via Illumina gene expression arrays using total RNA isolated from macro‐dissected prostate tissue sections from partially paired benign (n = 39) and cancer (n = 59) specimens. Expression data sets have been deposited in the NCBI Gene Expression Omnibus database as stated in Materials and methods. Gene expression levels are shown relative to the housekeeping gene hypoxanthine phosphoribosyltransferase (HPRT). Empty circles denote outliers excluded from the analysis. Statistically significant differences are indicated (ns, not significant where *P* > 0.05; **P < 0.01; ***P < 0.001).

It currently remains unclear which Nox isoforms are expressed in the prostate in vivo and whether they are involved in prostate carcinogenesis and progression. Thus, we queried the Human Protein Atlas repository (www.proteinatlas.org) for protein and RNA data relating to the expression of the different Nox/Duox isoforms. According to the repository Nox1 and Nox5 proteins are highly expressed in epithelial glandular cells of the prostate, whereas Nox2 and Nox4 are expressed at much lower levels in the prostatic epithelium with no detectable expression of Nox3 or Duox1–2 (summarized in Table 1). The repository does not permit accurate quantification of immunohistochemical staining intensity to reliably determine whether Nox/Duox protein levels differ between benign and cancer samples. Thus, we subsequently analyzed Nox/Duox mRNA expression levels on Illumina gene expression arrays using RNA isolated from macro‐dissected prostate tissue sections of partially paired benign versus cancer samples. Nox4 mRNA levels were significantly upregulated in PCa versus benign tissue, which is consistent with previous reports and reflects increased stromal Nox4 levels in PCa (Figure 1) 19, 20, 21. By contrast, Duox1 mRNA levels were significantly lower in PCa compared to benign prostate. A slight but significant decrease in Nox5 mRNA levels was also observed in PCa compared to benign specimens (Figure 1). Nox1–3 and Duox2 mRNA levels did not significantly differ between benign and malignant prostate tissues (Figure 1).

**Table 1:** Summary of Human Protein Atlas Entries for Expression and Localization of Nox/duox Isoforms in the Prostate

Nox isoform	Human Protein Atlas Information^a^
Nox1	High epithelial expression in benign and tumor cells
Nox2	Low expression in benign epithelial and tumor cells. Prominent stromal expression
Nox3	Only RNA data available, no detectable expression in prostate tissue or PC‐3 PCa cell line
Nox4	Low expression in benign epithelium and tumor cells, moderate staining of stroma
Nox5	High expression in benign epithelium and tumor cells
Duox1	No detectable expression in benign epithelium or tumor cells
Duox2	Only RNA data available, no detectable expression in prostate tissue or PC‐3 PCa cell line

^a^ Information derived from Human Protein Atlas repository (www.proteinatlas.org) version 12.

**Figure 2:**
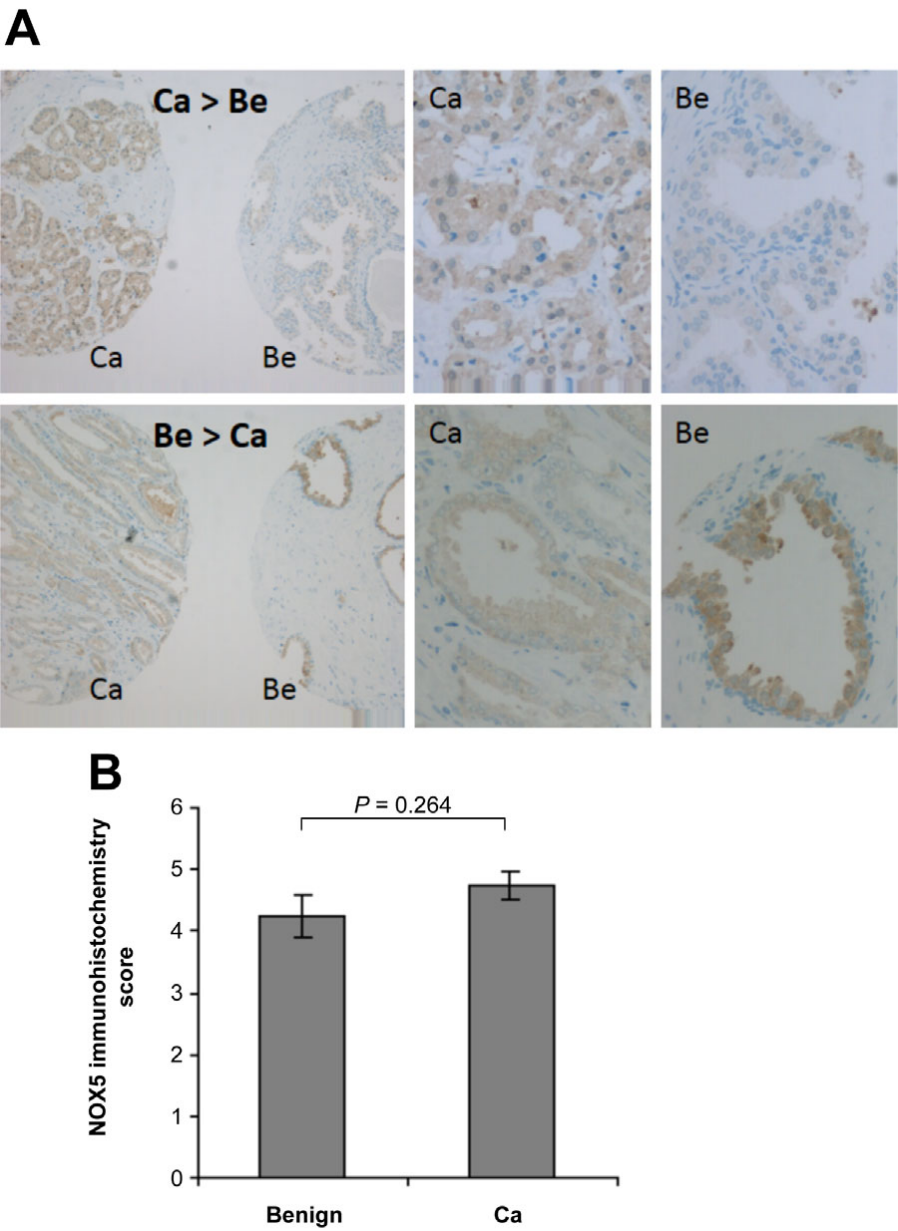
Nox5 is expressed in the glandular epithelium of the benign and malignant human prostate. A prostate TMA containing tumor and benign tissue cores of 48 patients (3 cancer, 1 benign tissue patient‐matched cores for each case) was immunostained with an anti‐Nox5 antibody. (A) Brown color indicates positive staining for Nox5, cell nuclei are counterstained in blue. Images are representative of a cancer (Ca) and patient‐matched benign (Be) tissue core, with one case exhibiting more intense Nox5 staining in the benign versus cancer core (Be > Ca) and one case exhibiting more intense Nox5 staining in the cancer versus benign core (Ca > Be). Left panel, magnification 10×. Enlarged sections of the cancer and benign cores (left panel) are shown (middle and right panels, respectively). (B) Nox5 immunoreactivity was scored as described in Material and methods. Forty‐four benign and 106 tumor tissue cores were finally evaluable. The histogram shows mean immunohistochemistry score (±SEM) for benign and cancer (Ca) tissue cores.

On the basis of their high expression level in luminal epithelial cells of the prostate and in tumor cells, the above data implicated Nox1 and Nox5 proteins in playing a physiologically important role in the benign and malignant prostatic epithelium. There are several reports documenting a potential role of Nox1 in prostate carcinogenesis 14, 18, 31, 32 but comparatively little is known about the function of Nox5 in prostate biology 12. Thus, subsequent efforts focused on investigating and characterizing Nox5 levels in the prostate. We next investigated Nox5 protein levels in benign prostate and PCa tissue via immunohistochemical staining of a TMA comprising 3 tumor and 1 benign tissue cores each for 48 patients using a highly specific Nox5 antibody (Figure 2 and Supplemental Figure S1). Consistent with the aforementioned data in the Human Protein Atlas, we observed epithelial‐specific expression of Nox5 in the majority of PCa specimens analyzed (Figure 2A). Benign epithelium as well as cancer cells displayed Nox5 immunoreactivity of varying intensities in different cases with varying tumor/benign ratios (Figure 2A). Nonetheless, the aforementioned slight but significant decrease in Nox5 mRNA levels (Figure 1) was not observed at the protein level and overall there was no significant difference in Nox5 protein levels between benign versus malignant tissue (Figure 2B).

### Nox5 Expression in Prostate Cancer Cell Lines

**Figure 3:**
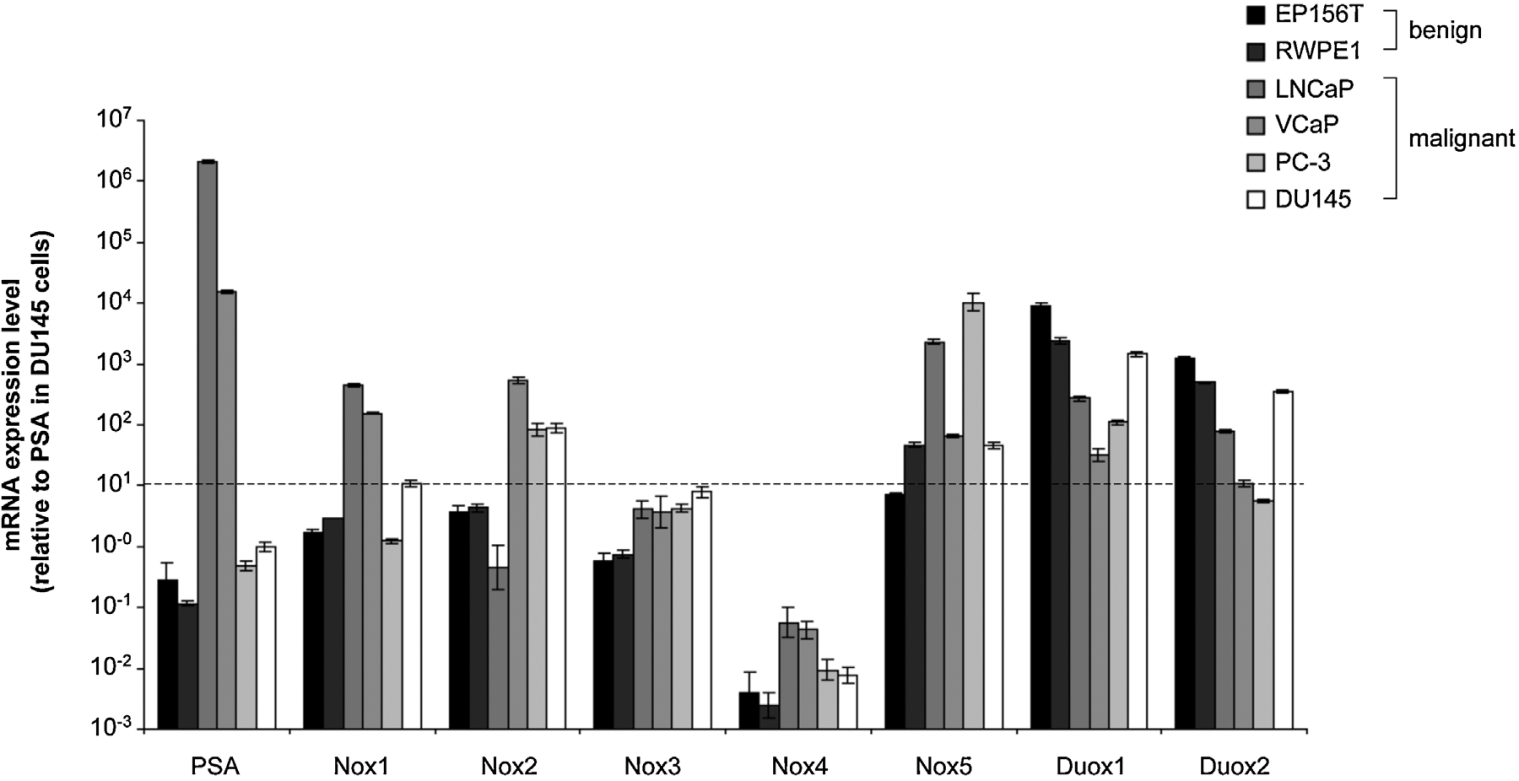
Expression of Nox/Duox isoforms in benign and malignant prostate cell lines. RNA from benign (EP156T and RWPE1) and malignant (DU145, LNCaP, PC‐3, and VCaP) prostate epithelial cell lines was prepared and relative expression levels of the indicated genes determined by qPCR. Values represent mean fold change (±SEM) in gene expression from triplicates in three independent experiments relative to EP156T cells and normalized against the housekeeping gene B2M. mRNA levels of PSA are shown as a positive control, as DU145 cells are considered to lack PSA expression 33, a threshold cut‐off score was arbitrarily set at a value of 10, below which the indicated genes were not considered to be expressed.

To identify an in vitro model system suitable for functional analyses of endogenous Nox5 in PCa we used qPCR to screen a panel of commonly used and well characterized benign (EP156T and RWPEI) and malignant (DU145, LNCaP, PC‐3, and VCaP) prostate epithelial cell lines for Nox5 expression (Figure 3). Published reports regarding the expression of Nox isoforms in PCa cell lines are highly inconsistent (summarized in Supplemental Table S1). Thus, we also examined expression levels of the remaining Nox/Duox isoforms (Figure 3). Prostate‐specific antigen (PSA) mRNA served as a positive control and was readily detected in LNCaP and VCaP cells but not in EP156T, RWPE1, DU145, or PC‐3 cells, as previously described 24, 33, 34. To facilitate comparison of Nox/Duox isoform expression across different cell lines, the PSA mRNA signal of DU145 cells, which are widely considered to lack PSA expression, was set as a zero‐expression level, and a cut‐off limit was set 10‐fold higher, above which Nox/Duox isoforms were considered to be expressed (Figure 3). In support of the biological relevance of this threshold, Western blotting of Nox1 revealed a faint but detectable band in lysates prepared from DU145 cells but not in those from PC‐3 cells (Supplemental Figure S2), which is in agreement with the qPCR data above (Figure 3). Under these cut‐off criteria, negligible levels of Nox3 and Nox4 mRNAs were detected across all cell lines tested, whereas Nox1 was considered to be expressed at low levels in DU145 cells and at higher levels in LNCaP and VCaP cells. By contrast, Nox2 was considered to be expressed in VCaP, PC‐3, and DU145 cells and Nox5 in RWPE1 and all four tested PCa cell lines with the highest levels in LNCaP and PC‐3 cells (Figure 3). Notably, mRNA levels of Nox1–5 were typically higher in PCa lines compared to the benign prostate cell lines tested, whereas the two Duox isoforms were expressed at higher levels in the benign cell lines than the PCa cell lines tested (Figure 3). Duox1 mRNA levels were readily detectable in EP156T, RWPE1, and DU145 cells whilst Duox2 was detectable at slightly lower levels in the same cell lines. Of the seven Nox/Duox isoforms, Nox5 was the most consistently expressed in PCa cells (Figure 3).

### Nox5‐Specific Silencing in PC‐3 Prostate Carcinoma Cells

**Figure 4:**
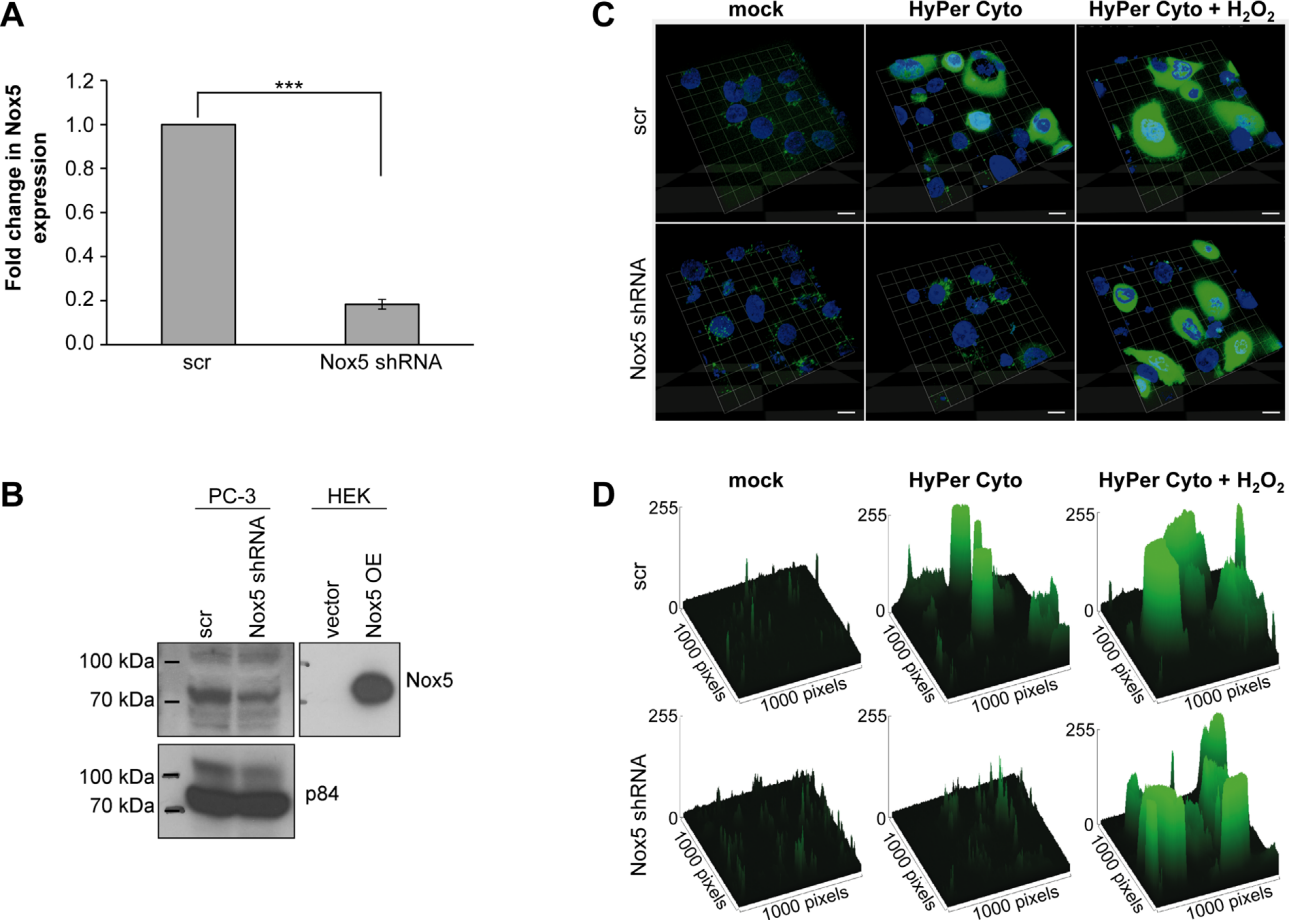
Isoform‐specific silencing of Nox5 reduces cytosolic H_2_O_2_ levels in PC‐3 cells. (A–D) PC‐3 cells were infected with lentiviral particles containing scrambled control (scr) or Nox5‐specific shRNA. Cells were selected using appropriate antibiotics and experiments performed on day 7 (A and B) or day 11 (C and D) after infection. (A) RNA was prepared and Nox5 expression analyzed by qPCR. Values represent mean fold change (±SEM) gene expression from three independent experiments relative to scr control and normalized against the housekeeping gene B2M. (B) Membrane lysates were prepared as described in Material and methods, loaded onto SDS/PAGE (HEK: 0,5 μg, PC‐3: 50 μg) and analyzed by Western blot using the antibodies indicated. HEK cells transfected to transiently overexpress Nox5 (Nox5 OE) were used as positive control. Nuclear protein p84 was used as loading control. (C and D) Cells were transfected with a plasmid expressing the untagged (i.e., cytoplasmic) pHyPer‐dCyto reporter protein and analyzed by live cell confocal microscopy 24 h thereafter. Fluorescence of the H_2_O_2_‐sensitive HyPer protein is depicted in green and nuclei counterstained with Höchst 33259 are shown in blue. Cells pre‐treated for 30 min with H_2_O_2_ (250 μM) served as positive control. Images shown are 3D graphs of z‐stacks of 5 planes with a spacing of 1 μm. Magnification 63×. Scale bar represents 10 μm. (D) Images represent surface blots of images shown in (C) where peak height correlates to signal intensity. Images were illustrated using Image J software. (B and D) Images are representative of three independent experiments.

We next focused on investigating the potential function of Nox5 in PCa cells. To this end, Nox5 was silenced in PC‐3 cells by infection with lentiviruses expressing shRNA specifically targeting Nox5 or control (scrambled) shRNA. This resulted in a significant reduction in Nox5 expression both at the mRNA and protein level (Figure 4A and B). Depletion of Nox5 had no pronounced effect on the expression levels of Nox1 or Nox2 (Supplemental Figure S3A). However, Nox4 mRNA levels were slightly elevated in Nox5‐depleted PC‐3 cells, although Nox4 protein remained below the detection level (Supplemental Figure S3A and B). This inability to detect Nox4 protein was not due to technical failure since endogenous Nox4 could be readily detected in normal human umbilical vein endothelial cells as described 6 (Supplemental Figure S3B). Thus, it was concluded that the slight increase in Nox4 mRNA levels in Nox5‐depleted PC‐3 cells is without functional significance and that the Nox5 shRNA employed leads to isoform‐specific silencing.

Using the H_2_O_2_‐sensitive fluorescent protein HyPer reporter protein system (for details see Materials and methods), we monitored cytosolic and mitochondrial hydrogen peroxide levels. With the untagged (i.e., cytoplasmic) protein HyPer‐dCyto, we observed a significant decrease in cytosolic H_2_O_2_ levels in Nox5‐depleted cells (Figure 4C and D), whereas mitochondrial H_2_O_2_ levels, probed with the mitochondrial‐targeted protein HyPer‐dMito were unaffected by Nox5 depletion (Supplemental Figure S4). The reduced levels of cytosolic H_2_O_2_ in Nox5‐depleted cells were not due to differences in transfection efficiency of the HyPer plasmid as H_2_O_2_ treatment of Nox5 shRNA treated cells resulted in levels of fluorescence comparable to H_2_O_2_ treated scr control cells (Figure 4C and D). Thus, these data suggest that Nox5 is the major source of cytosolic H_2_O_2_ in PC‐3 cells under basal conditions and are consistent with the predominant expression of Nox5 compared to other Nox/Duox isoforms in this cell line (Figure 3).

### Nox5 Contributes to Proliferation and Survival of Prostate Carcinoma Cells

**Figure 5:**
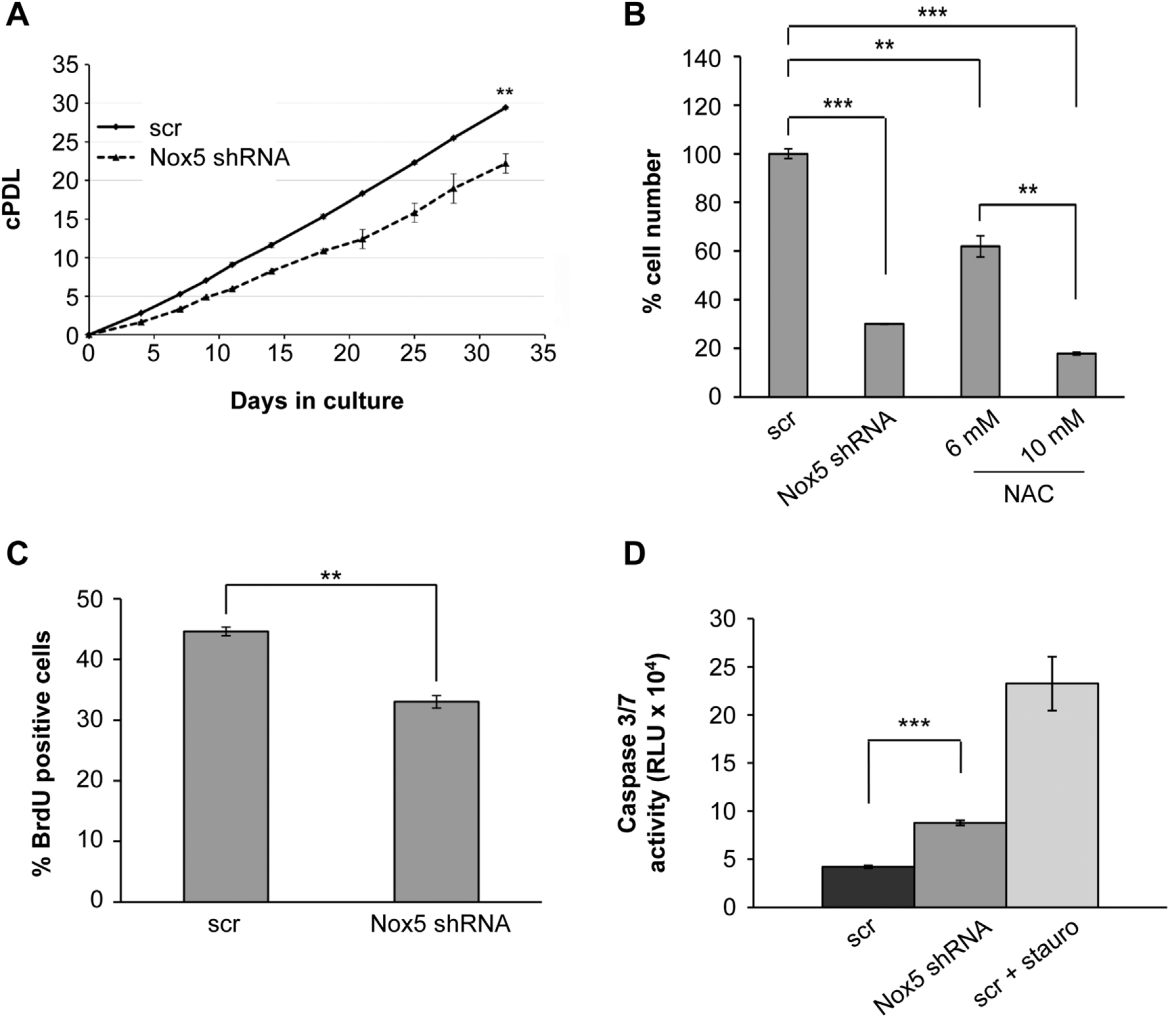
Isoform‐specific silencing of Nox5 impairs proliferation and survival of PC‐3 prostate carcinoma cells. (A–D) PC‐3 cells were infected with lentiviral particles containing scrambled control (scr) shRNA or Nox5‐specific shRNA and selected using appropriate antibiotics. (A) Cells were passaged at regular intervals, counted and cell numbers used to establish a growth curve, which is displayed as cumulated population doublings (cPDL) and calculated as described in Materials and methods. Values represent the mean of three replicates from five independent experiments (±SEM). (B) In parallel to Nox5‐mediated silencing, PC‐3 cells were treated with the indicated concentrations of the ROS scavenger N‐acetylcysteine (NAC). All cells were passaged at regular intervals, counted at 9 days following NAC treatment or lentiviral infection and normalized to cell numbers in scr control treated cells, which were set to 100%. Values represent mean of triplicates (±SEM) from two independent experiments. (C) Cell proliferation was determined 7 days after lentiviral infection and the number of BrdU positive cells determined as described in Materials and methods. Values represent mean of three independent experiments (±SEM). (D) Induction of apoptosis after Nox5 knockdown was determined by measuring Caspase 3/7 activity. Cells pre‐treated with 1 μM staurosporine (stauro) for 4.5 h served as positive control. Mean values (±SEM) from quadruplicate wells of four independent experiments are shown. (A–D) Statistical significance is shown (**P < 0.01; ***P < 0.001).

Nox‐derived ROS are implicated in regulating numerous essential biological processes, including proliferation and apoptosis 35, 36. Thus, we next examined whether Nox5 knockdown led to changes in cell proliferation or survival. Indeed, Nox5 silencing resulted in a significant decrease in the rate of proliferation in PC‐3 cells (Figure 5A–C). Importantly, similar effects were observed in the Nox5‐positive PCa cell line LNCaP with only a weak but non‐significant trend in DU145 PCa cells, which express only very low levels of Nox5 (Supplemental Figure S5 and Figure 3). Thus, Nox5 depletion has similar effects on cell proliferation across three different PCa cell lines. The decrease in PC‐3 cell proliferation could be mimicked by ROS depletion using the antioxidant N‐acetylcysteine (NAC) (Figure 5B). Moreover, Nox5 silencing led to a significant increase in apoptotic cell death as indicated by enhanced activation of caspases 3 and 7 (Figure 5D). Together these observations suggest that Nox5‐derived ROS contributes significantly to the proliferation and survival of PC‐3 PCa cells.

### Nox5 Depletion Impairs JNK Phosphorylation and Reduces PKC‐Zeta Protein Levels

**Figure 6:**
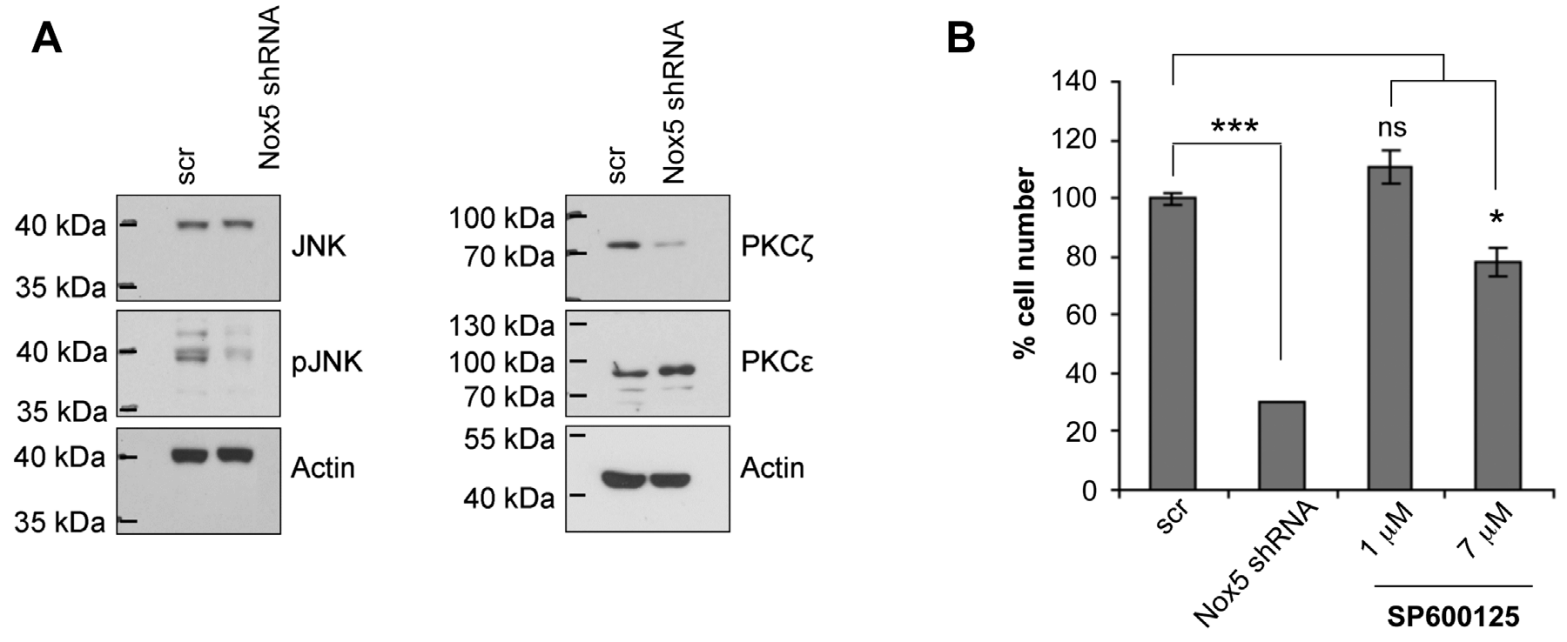
Nox5 silencing impairs JNK phosphorylation and reduces PKC zeta protein levels. (A and B) PC‐3 cells were infected with lentiviral particles containing scrambled control (scr) or Nox5‐specific shRNA. Cells were selected using appropriate antibiotics and experiments performed 7 days after infection. (A) Total cell lysates were prepared from scr control and Nox5‐depleted PC‐3 cells and analyzed by Western blotting using the antibodies indicated. Images are representative of two independent experiments. (B) In parallel to Nox5‐mediated silencing, PC‐3 cells were treated for 7 days with the indicated concentration of JNK inhibitor SP600125, passaged at regular intervals and cell numbers counted. Values represent mean cell number (±SEM) relative to scr control, which was set to 100% from triplicate wells. Significance is indicated (ns, *P* < 0.05; *P < 0.05; ***P < 0.001).

We next sought to identify the molecular mechanism(s) underlying the pro‐proliferative effect of Nox5 in PCa cells. As Nox‐derived ROS primarily mediate their signaling functions via oxidative modification of redox‐sensitive kinases and phosphatases 37, 38, we screened a number of different signaling intermediates. Nox5 depletion clearly reduced phosphorylation of c‐Jun N‐terminal kinase 1/3 (JNK1/3) and also reduced protein levels of PKC‐zeta (PKCζ) (Figure 6A). In contrast, signaling through AKT, p38 and ERK1/2, which are also implicated in cell proliferation, was not detectably affected by Nox5 depletion (Supplemental Figure S6 and data not shown). Similar to Nox5 depletion and consistent with a role of JNK signaling in the proliferation of PC‐3 cells, the JNK inhibitor SP600125 significantly inhibited proliferation of PC‐3 cells (Figure 6B). These data indicate that Nox5‐derived ROS are required for specific signaling pathways in PC‐3 cells.

## DISCUSSION

Elevated ROS and NADPH oxidases have been implicated in playing a role in prostate carcinogenesis for some time 12, 13, 14, 18, 23, 32, 39. However, the identity and pro‐tumorigenic mechanism of action of the Nox enzyme(s) involved remain largely unknown. According to the Human Protein Atlas, the benign and malignant prostatic epithelium expresses high levels of Nox1 and Nox5 proteins, suggesting they may be important in prostate physiology. Similarly, immunohistochemical staining of a human prostate TMA revealed distinct Nox5 expression in epithelial cells of prostatic glands, indicating that Nox5 is clearly expressed in the human prostate. Expression analyses revealed that Nox5 is the most consistently expressed isoform of the Nox/Duox family in the PCa cell lines examined. Moreover, we found significantly higher levels of Nox5 mRNA in LNCaP and PC‐3 cells compared to benign cell lines, which is in agreement with previous studies demonstrating that PCa cell lines exhibit higher levels of extra‐mitochondrial ROS than benign prostate cells 13. Isoform‐specific shRNA‐mediated silencing of Nox5 impaired proliferation of PCa cell lines that express readily detectable levels of Nox5 (namely PC‐3 and LNCaP) but had only weak and non‐significant effect on DU145 cells, which express only very low levels of Nox5. In addition to impaired proliferation, Nox5 depletion also increased apoptosis of PC‐3 cells and was associated with reduced phosphorylation of JNK1/3 and reduced protein levels of PKCζ. Collectively, these data indicate that Nox5‐derived ROS play a critical role in regulating the proliferation and survival of PCa cells. Together with the observation that Nox5 is expressed in clinical PCa, these data suggest that Nox5 may represent a potential therapeutic target for the treatment of PCa.

This study primarily focused on investigating Nox5. However, inconsistent and incomplete reports in the literature regarding the expression of Nox/Duox isoforms in different PCa cell lines prompted us to perform a systematic analysis of Nox/Duox isoform mRNA levels in commonly used benign and malignant prostate cell lines. Compared to Nox5, lower levels of Nox1 were detected in LNCaP and VCaP cells, the latter also expressing detectable levels of Nox2 mRNA, which is additionally expressed in PC‐3 and DU145 cells. These findings are somewhat different to studies published by others. For example, in contrast to previous studies 13, 18, we were unable to detect substantial Nox4 expression in any of the epithelial prostate cell lines analyzed. This finding is supported by protein data from the Human Protein Atlas, which shows only very weak epithelial Nox4 staining but a much higher level of staining in the prostatic stroma, which is consistent with published studies demonstrating a role of elevated stromal Nox4 in PCa 19, 21. It is likely that the differences between this and already published studies are due to methodological and cellular model differences, including a lack of robust Nox isoform‐specific antibodies, use of different detection systems and divergent cell (sub) lines due to different culture conditions in different laboratories. Nonetheless, our observation that Nox5 is abundantly expressed in LNCaP and PC‐3 PCa cell lines is supported by immunohistochemical data using an extensively validated antibody and supported by data from the Human Protein Atlas, which together demonstrate that Nox5 is expressed in the benign and malignant prostatic epithelium in vivo and at abundant levels in clinical PCa.

Nox5 has been implicated in several malignancies, however its role in tumor cell biology remains unclear. In fact, compared to other Nox isoforms the functional significance of Nox5 is considerably less well understood. This is due in part to a lack of reliable antibodies against Nox5 but also because a Nox5 homolog is not present in the rodent genome and due to a lack of studies of Nox5 in human tissues 40. Using a Nox5 antibody validated by stringent biochemical criteria, we demonstrate herein that Nox5 is abundantly expressed in both benign prostate epithelia and in clinical PCa, although not at statistically different levels between patient‐matched benign and malignant prostate tissues. To our knowledge, this is the first quantification of Nox5 protein levels in human benign and malignant prostate tissue. These data are consistent with a recent report by Antony et al. in which Nox5 expression was detectable in several human cancers, including PCa 41. Unfortunately however, the authors did not include Nox5 immunohistochemical staining of benign prostate in their study, thus they were unable to draw any conclusions as to whether Nox5 protein levels differ between the benign and malignant prostate 41.

In vivo data presented herein indicate that whilst Nox5 mRNA levels were slightly but significantly lower in PCa compared to benign specimens, there is no significant difference at the protein level (Figures 1 and 2). This is in contrast to the higher expression levels (both at mRNA and protein level) of Nox5 in PCa cell lines (LNCaP and PC‐3) compared to benign cell lines (RWPE1 and EP156T) (Fig. 3). This apparent discrepancy may reflect adaptation of the LNCaP and PC‐3 cell lines to in vitro growth conditions. However, given the functional data herein that indicate Nox5 plays a critical role in regulating cell proliferation and survival, it is plausible that immortalization of the RWPE1 and EP156T cell lines via human papillomavirus 18 or h‐TERT, respectively bypasses the need for Nox5 in terms of proliferation and survival resulting in its down‐regulation in these benign cell lines. Further studies are required to address this issue. Nonetheless, similar to the high expression of Nox5 in clinical PCa, the abundant expression of Nox5 in LNCaP and PC‐3 cell lines makes them ideal tools for functional investigation of Nox5 in PCa.

Although we observed no significant difference in total protein levels of Nox5 during prostate tumorigenesis, one should bear in mind that Nox5 is subject to posttranslational activation 42, 43, 44, 45, 46. Thus, comparable protein abundance in benign epithelial and cancer cells does not exclude enhanced activity and ROS production in tumor tissue. For example, Nox5 activity is strongly enhanced by binding of Ca^2+^ to the N‐terminal region of Nox5 42. In this respect it may be noted that a large body of evidence suggests that increased calcium intake is associated with enhanced PCa risk 47. Moreover, the Ca^2+^ channel TRPV6, which is overexpressed in PCa and correlates with tumor progression and poor prognosis, is also expressed at higher levels in the Nox5‐positive PCa cell lines PC‐3 and LNCaP compared to normal and benign epithelial cells 48, 49, 50, 51. Thus, it is possible that elevated Ca^2+^ signaling in PCa may lead to an increase in Nox5 activity. In addition to activation by Ca^2+^, Nox5 activity is also enhanced by (i) PKC‐ and ERK1/2‐mediated phosphorylation, which increases Nox5 sensitivity to Ca^2+^ and thereby enables ROS production at lower Ca^2+^ levels; and (ii) by interaction with membrane phospholipids 40, 43, 44, 45, 46. In particular, PKCα directly phosphorylates and activates Nox5 43. Notably, PKCα plays a key role in the regulation of downstream oncogenic molecules in PCa and its activation is required for the survival and growth of androgen‐independent human PCa cells, including PC‐3 52, 53, 54, 55. Thus, it is likely that Nox5 activity is elevated in PCa, however verification by quantifying Nox5 activity in benign and malignant prostate tissues is required.

Data presented herein indicate that inactivation of Nox5 gene expression by shRNA‐mediated silencing reduces both proliferation and survival of androgen‐dependent (LNCaP) as well as androgen‐independent (PC‐3) cells. This is consistent with reports that Nox5 knockdown in the PCa cell line DU145 and esophageal adenocarcinoma cell line SEG1‐EA also result in attenuated proliferation and increased apoptosis 12, 56. Together these findings support the notion that Nox5 activation and subsequent ROS production contribute to prostate tumor cell malignancy. The precise molecular mechanism by which Nox5 contributes to the regulation of cell proliferation and survival of PCa cells are not fully understood. However, we observed that Nox5 depletion reduced PKCζ protein levels and impaired JNK1/3 phosphorylation, the latter observation being consistent with reports showing that JNK phosphorylation can be induced by Nox‐derived ROS 19, 57. Moreover, elevated expression of PKCζ is a characteristic of PCa and required for the malignant potential of PC‐3 PCa cells 58, 59. As PKCζ is critical for JNK activation and translocation of NFκB resulting in pro‐proliferative and pro‐survival pathways, it seems plausible that decreased JNK1/3 phosphorylation, proliferation and increased apoptosis upon Nox5 silencing are direct consequences of decreased PKCζ protein levels 60, 61. PKCζ appears to influence tumorigenesis via different molecular pathways 62. However, in the context of Nox5 it is noteworthy that ROS can induce nuclear translocation of PKCζ in mouse embryonic fibroblasts and HeLa cells, where it has been shown to regulate cell viability by suppressing apoptotic cell death 62, 63, 64. Such a mechanism may account for the increase in apoptosis upon Nox5 knockdown in PC‐3 cells observed in this study. Moreover, PKCζ is activated by the sphingolipid‐derived second messenger molecule ceramide, which forms membrane raft‐derived signaling platforms that activate ROS‐generating systems, including Nox enzymes 65. Ceramide production as well as the formation of these ceramide‐enriched membrane platforms is also induced by ROS 65. Thus, it is possible that an amplifying feedforward loop exists whereby Nox5‐derived ROS on the one hand induce expression of PKCζ, which in turn phospho‐activates JNK leading to cell proliferation and survival, and on the other hand promote ceramide production and membrane raft platform formation further increasing Nox5 and PKCζ activity.

In summary, in vitro and in vivo data presented herein establish a role of Nox5 in the prostatic epithelium and suggest that Nox5‐derived ROS act as a regulator of epithelial cell proliferation and survival in this tissue, most likely by modulating the activity and expression of PKCζ and JNK1/3. In addition to PCa, Nox5 is also expressed at elevated levels in breast, colon, lung, brain, and ovary cancer, malignant melanoma and non‐Hodgkin lymphoma and has also been implicated in the development of esophageal adenocarcinoma 40, 41, 66. Thus, these data are of potential interest not only for researchers in the field of prostate cancer research but also of broad interest for other cancer disciplines.

## Supporting information

Additional supporting information may be found in the online version of this article at the publisher's web‐site.

Supporting Fig. S1.

Supporting Fig. S2.

Supporting Fig. S3.

Supporting Fig. S4.

Supporting Fig. S5.

Supporting Fig. S6.

Supporting Data.
