# Supplementary figures and images for: ROS signaling by NADPH oxidase 5 modulates the proliferation and survival of prostate carcinoma cells
Source: Mol Carcinog. 2015 Jan 5;55(1):27–39. doi: 10.1002/mc.22255 (PMC4949723; doi:10.1002/mc.22255)

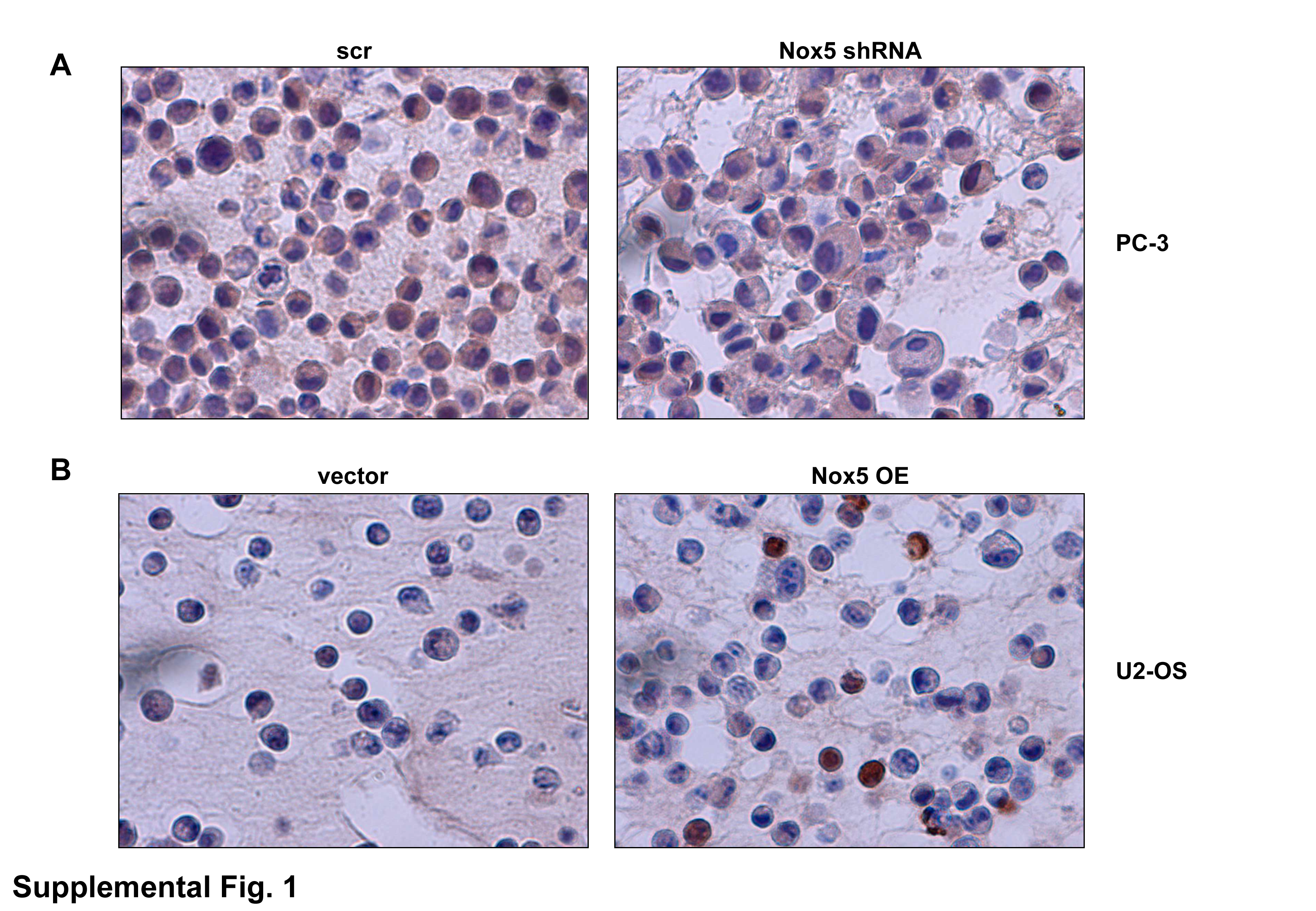

Supplement: Supplementary file 1 — Supporting Fig. S1. [file MC-55-27-s001.tif]

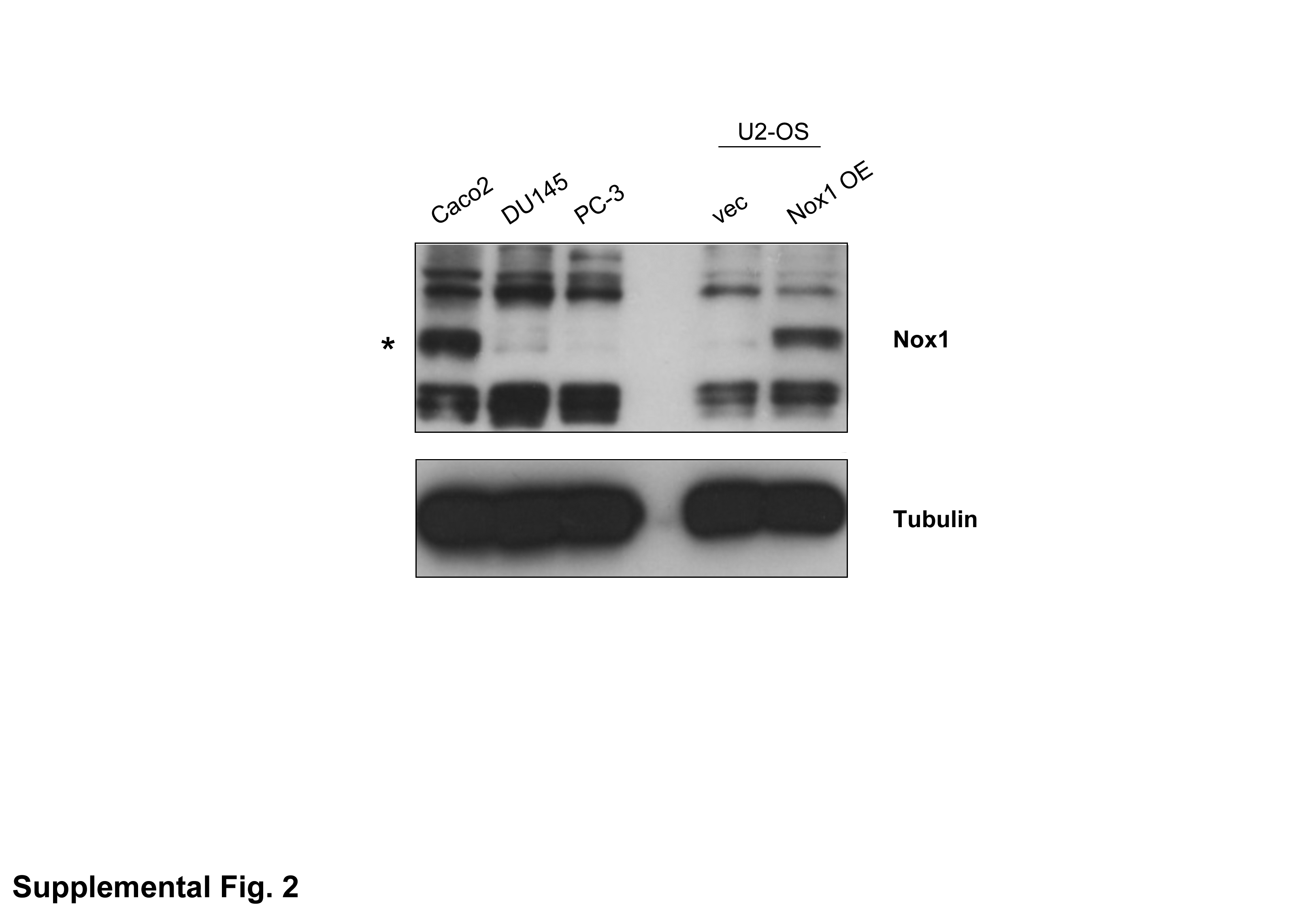

Supplement: Supplementary file 2 — Supporting Fig. S2. [file MC-55-27-s002.tif]

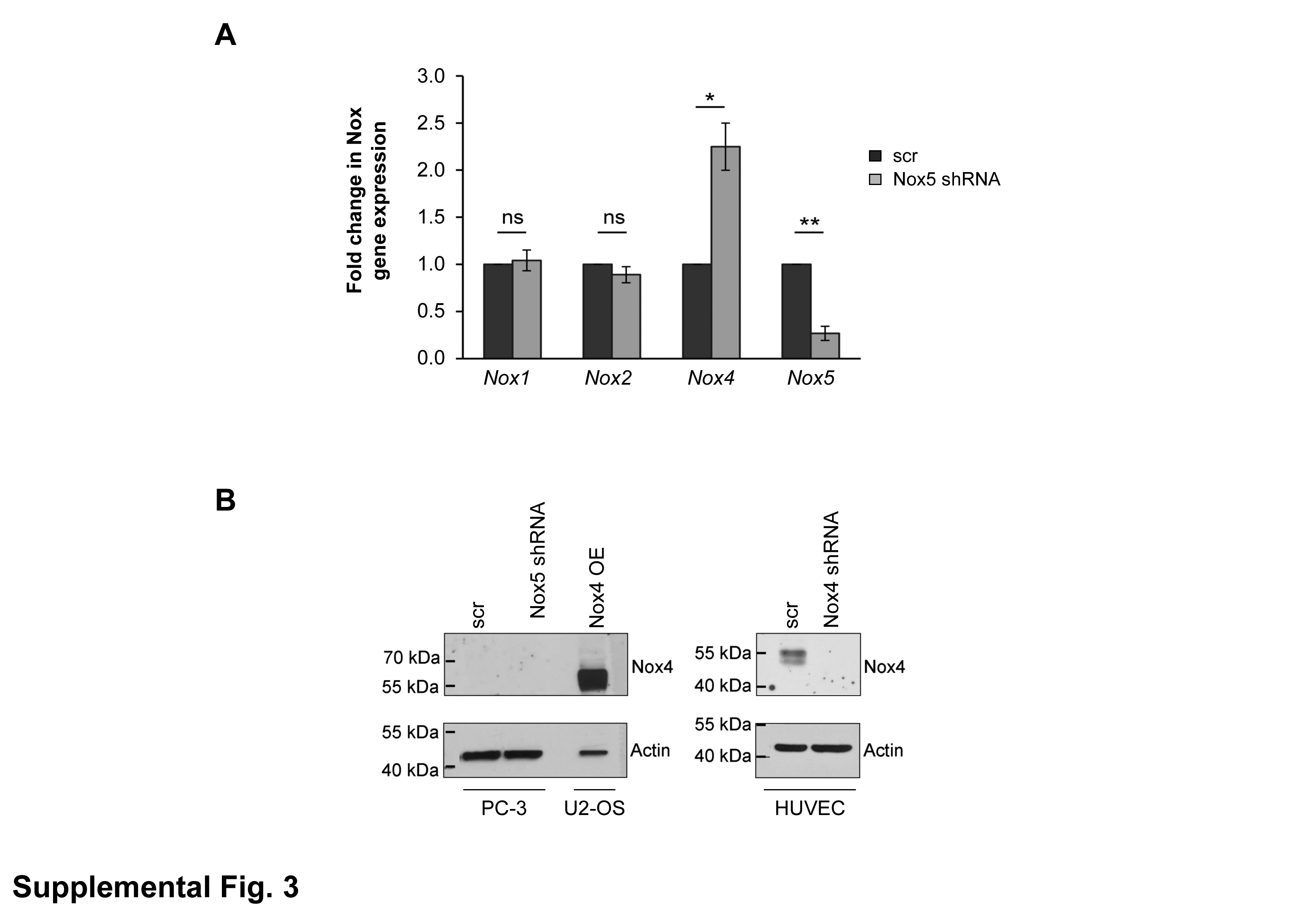

Supplement: Supplementary file 3 — Supporting Fig. S3. [file MC-55-27-s003.tif]

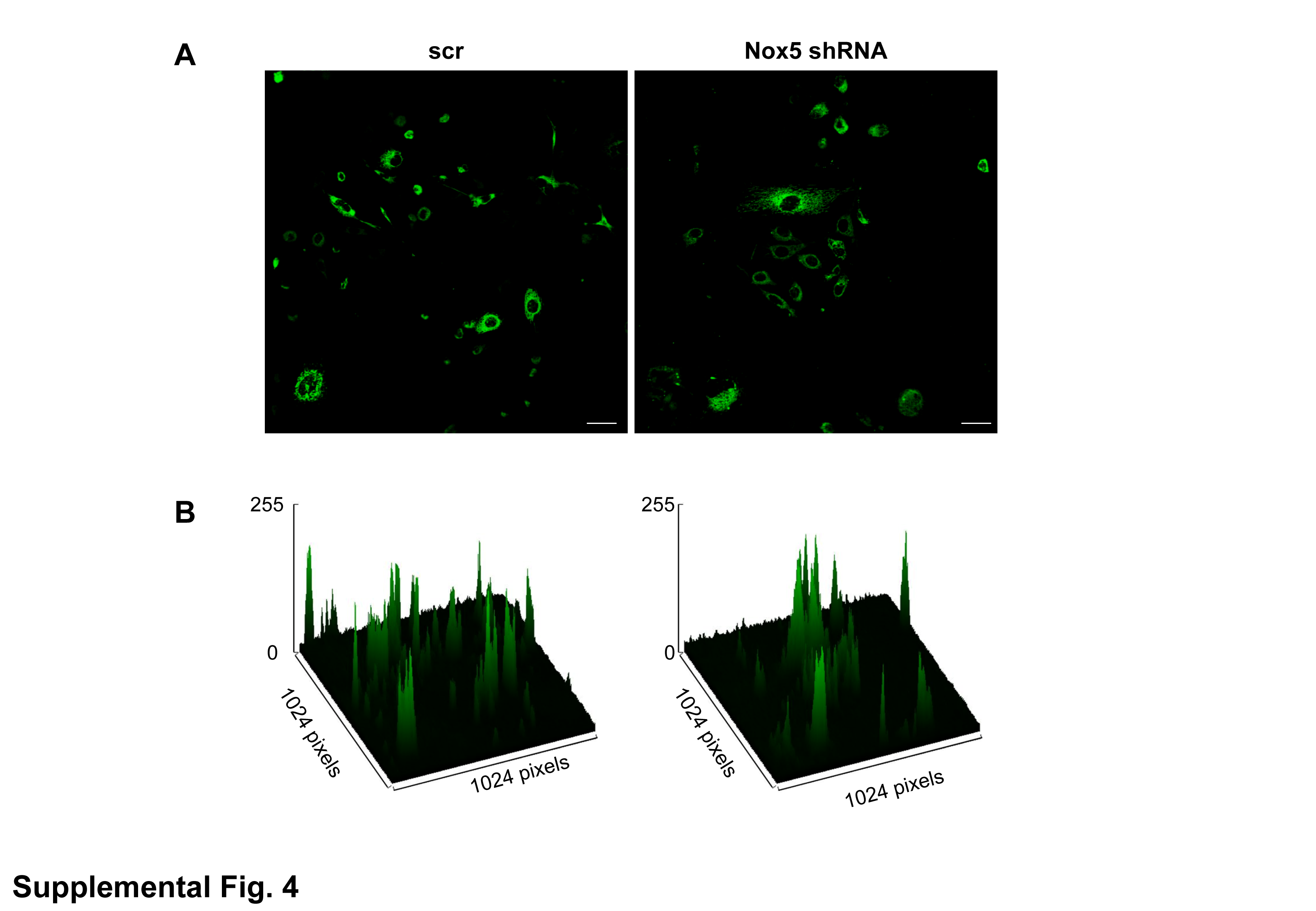

Supplement: Supplementary file 4 — Supporting Fig. S4. [file MC-55-27-s004.tif]

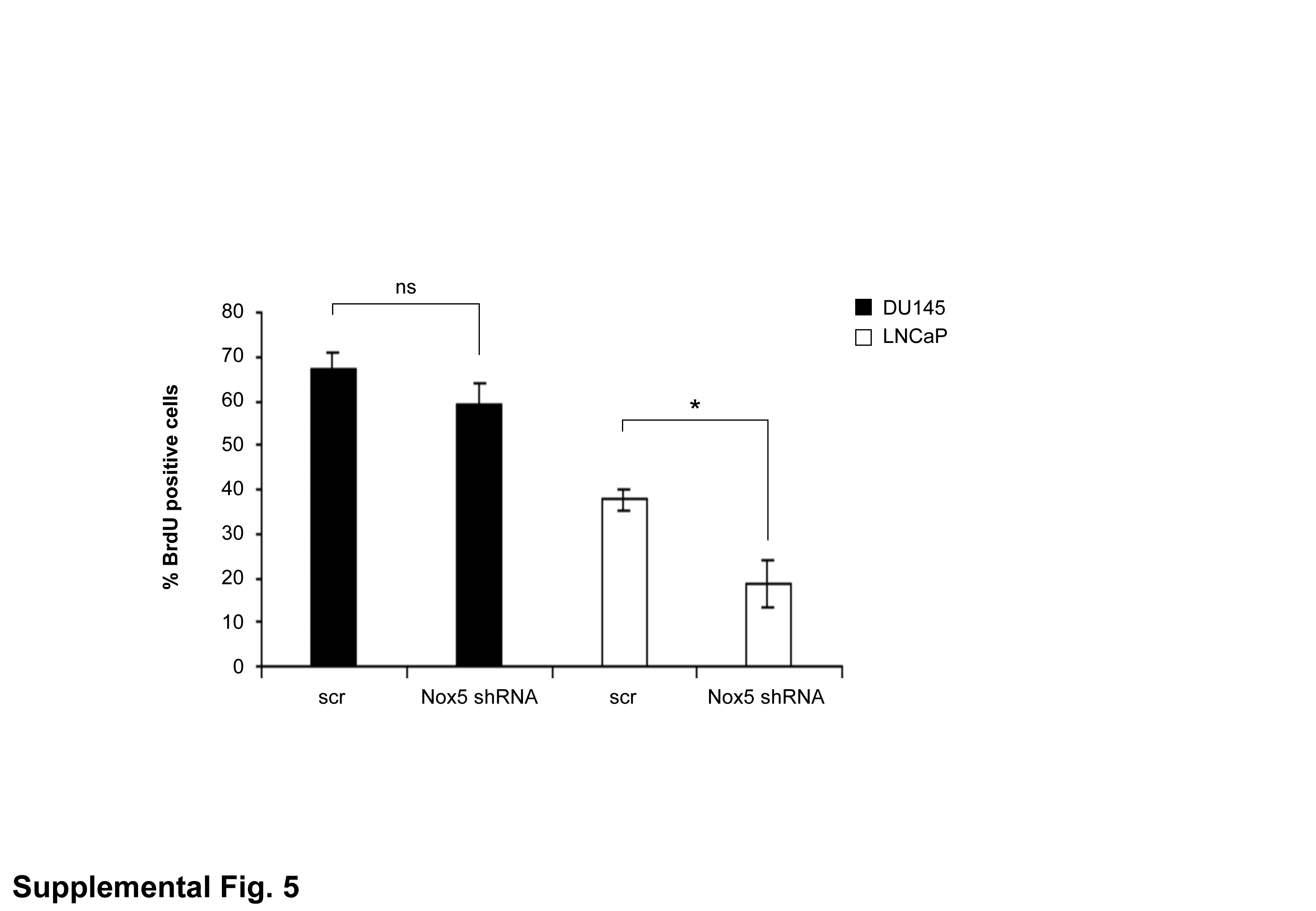

Supplement: Supplementary file 5 — Supporting Fig. S5. [file MC-55-27-s005.tif]

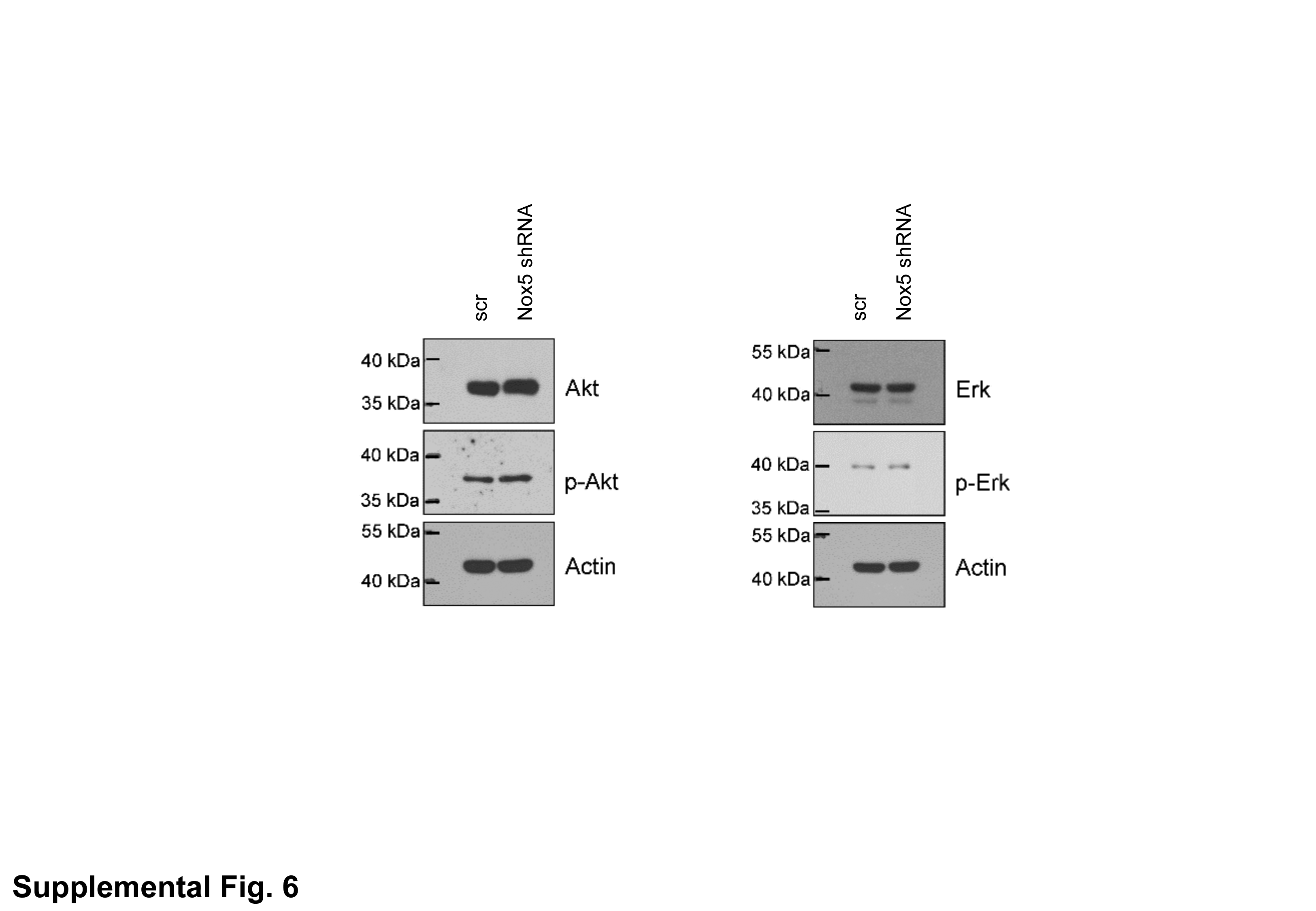

Supplement: Supplementary file 6 — Supporting Fig. S6. [file MC-55-27-s006.tif]
