# Supplementary material for: ROS signaling by NADPH oxidase 5 modulates the proliferation and survival of prostate carcinoma cells
Source: Mol Carcinog. 2015 Jan 5;55(1):27–39. doi: 10.1002/mc.22255 (PMC4949723; doi:10.1002/mc.22255)
Supplement: Supplementary file 7 — Supporting Data. [file MC-55-27-s007.doc]

**Supplemental Information**

**Supplemental Table 1 Overview of published studies reporting Nox enzyme mRNA expression in prostate cancer cell lines**

| **Reference** | **Nox isoform** | **Prostate cancer cell line** | | |
| --- | --- | --- | --- | --- |
|  |  | **PC3** | **LNCaP** | **DU145** |
| [12] | Nox1 | n/a | ✓ | - |
|  | Nox2 | n/a | n/a | n/a |
|  | Nox3 | n/a | n/a | n/a |
|  | Nox4 | n/a | - | - |
|  | Nox5 | n/a | ✓ | ✓ |
| [13] | Nox1 | - | - | - |
|  | Nox2 | ✓ | ✓ | ✓ |
|  | Nox3 | - | - | - |
|  | Nox4 | ✓ | ✓ | ✓ |
|  | Nox5 | ✓ | ✓ | - |
| [18] | Nox1 | ✓ | n/a | ✓ |
|  | Nox2 | ✓ | n/a | ✓ |
|  | Nox3 | - | n/a | - |
|  | Nox4 | ✓ | n/a | ✓ |
|  | Nox5 | - | n/a | - |
| [15] | Nox1 | ✓ | n/a | n/a |
|  | Nox2 | ✓ | n/a | n/a |
|  | Nox3 | - | n/a | n/a |
|  | Nox4 | ✓ | n/a | n/a |
|  | Nox5 | ✓ | n/a | n/a |
| [22] | Nox1 | ✓ | n/a | n/a |
|  | Nox2 | - | n/a | n/a |
|  | Nox3 | - | n/a | n/a |
|  | Nox4 | - | n/a | n/a |
|  | Nox5 | - | n/a | n/a |
| Höll et al., this study | Nox1 | - | ✓ | ✓ |
|  | Nox2 | ✓ | - | ✓ |
|  | Nox3 | - | - | - |
|  | Nox4 | - | - | - |
|  | Nox5 | ✓ | ✓ | ✓ |
| Detectable expression (✓) versus no detectable expression (-) of Nox isoforms at the mRNA level in PCa cell lines according to the available literature (references are cited). * No data were shown in this study but a statement in text indicated that only Nox1 could be detected. Nox isoforms or cells line not investigated by a particular study are indicated n/a (not applicable). | | | | |

**Supplemental Figure Legends**

**Supplemental Fig. 1 Nox5 antibody specificity**

Immunohistochemical staining of Nox5 in cell clots prepared from (A) PC-3 cells infected with lentiviral particles containing scrambled control (scr) or Nox5-specific shRNA and selected using appropriate antibiotics or (B) the Nox5 negative osteosarcoma cell line U2-OS, which was transfected to transiently overexpress Nox5 (Nox5 OE) or empty vector control (vector). Brown color indicates positive staining for Nox5. Cell nuclei were counterstained in blue. Original magnification 40x.

**Supplemental Fig. 2 Nox1 is detectable at the protein level in DU145 but not in PC-3 PCa cells**

Western blotting of Nox1 in cell lysates prepared from DU145 or PC-3 PCa cell lines as well as from Caco2 cells, which served as a positive control. The osteosarcoma cell line U2-OS, which lacks detectable Nox1 protein, was transiently transfected to overexpress Nox1 (Nox1 OE) or with empty vector (vec) as a further positive and negative control, respectively. Immunostaining of α-tubulin served as loading control. Images are representative of three independent experiments. *, denotes specific band corresponding to Nox1 protein.

**Supplemental Fig. 3 Specificity of Nox5 shRNA-mediated silencing in PC-3 cells**

PC-3 cells were infected with lentiviral particles containing scrambled control (scr) or Nox5-specific shRNA. Cells were selected using appropriate antibiotics and experiments performed 7 days after infection. (A) RNA was prepared and expression of Nox1, Nox2, Nox4 and Nox5 mRNA determined by qPCR. Values represent mean fold change (± SEM) in gene expression from 3 independent experiments relative to scr control and normalized against the housekeeping gene B2M. Significance is indicated (ns, not significant where *P* < 0.05; *, *P* < 0.05; **, *P* < 0.01). (B) Membrane lysates were prepared as described in Material and Methods, loaded onto SDS/PAGE and analyzed by Western blot using anti-Nox4 rabbit monoclonal 27-6 antibody. U2-OS cells transfected to transiently overexpress Nox4 (Nox4 OE) served as positive control. As an additional control for detection of endogenous Nox4 by the anti-Nox4 rabbit monoclonal 27-6 antibody, Western blots of membrane lysates from HUVEC cells infected with lentiviral particles containing scr or Nox4-specific shRNA are shown.

**Supplemental Fig. 4 Nox5 depletion has no effect on mitochondrial H_2_O_2_ production**

(A-B) PC-3 cells were infected with lentiviral particles containing scrambled control (scr) or Nox5-specific shRNA. Cells were selected using appropriate antibiotics and 11 days after infection were subsequently transfected with a plasmid expressing the tagged (i.e. mitochondrial) pHyPer-dMito reporter protein and analyzed by live cell confocal microscopy 24 h thereafter. (A) Fluorescence of the H_2_O_2_-sensitive HyPer protein is depicted in green. Images shown are 3D graphs of z-stacks of 5 planes with a spacing of 1 µm. Magnification 20x. Scale bar represents 20 µm. (B) Images represent surface blots of images shown in (A) where peak height correlates to signal intensity (given in arbitrary units from 0 to 255). Images were illustrated using ImageJ software.

**Supplemental Fig. 5 Effects of Nox5 depletion on cell proliferation in PCa cell lines**

The indicated PCa cell lines were infected with lentiviral particles containing scrambled control (scr) or Nox5-specific shRNA. Cells were selected using appropriate antibiotics. Fourteen days after infection cell proliferation was analyzed by BrdU staining as described in Materials and methods. Values represent mean number of BrdU positive cells (± SEM) expressed as percent from triplicates. Significance is indicated (ns, not significant where *P* > 0.05; *, *P* < 0.05).

**Supplemental Fig. 6 Effects of Nox5 depletion on cellular signal transduction pathways**

PC-3 cells were infected with lentiviral particles containing scrambled control (scr) or Nox5-specific shRNA. Cells were selected using appropriate antibiotics. Seven days after infection total cell lysates were isolated and analyzed by Western blotting using the antibodies indicated.
